# Population Pharmacokinetics of Radotinib in Healthy Volunteers and Patients with Chronic Myeloid Leukemia

**DOI:** 10.3390/ph18111705

**Published:** 2025-11-10

**Authors:** Minseo Kang, Jiwon Kim, Yerin Lee, Jae Soo Shin, Min Soo Park, Qian Jiang, Eun Kyoung Chung, Jangik I. Lee

**Affiliations:** 1 College of Pharmacy and Research Institute of Pharmaceutical Sciences, Seoul National University, Seoul 08826, Republic of Korea; yh101011@snu.ac.kr (M.K.); candycandy98@snu.ac.kr (J.K.); dldpfls98@snu.ac.kr (Y.L.); 2 IL-YANG Pharmaceutical Co., Ltd., Yongin 17096, Republic of Korea; jsshin@ilyang.co.kr; 3 Department of Clinical Pharmacology, Severance Hospital, Yonsei University College of Medicine, Seoul 03722, Republic of Korea; minspark@yuhs.ac; 4 Peking University People’s Hospital, Peking University Institute of Hematology, National Clinical Research Center for Hematologic Disease, Beijing 100044, China; jiangqian@medmail.com.cn; 5 Departments of Pharmacy and Regulatory Science, College of Pharmacy, Kyung Hee University, Seoul 02447, Republic of Korea; 6 Institutes of Regulatory Innovation Through Science (IRIS) and Integrated Pharmaceutical Sciences, Kyung Hee University, Seoul 02447, Republic of Korea

**Keywords:** radotinib, nonlinear mixed-effects modeling, population pharmacokinetics, tyrosine kinase inhibitor, chronic myeloid leukemia

## Abstract

**Background/Objectives**: Radotinib is a second-generation tyrosine kinase inhibitor (TKI) that has been used for treatment of chronic myeloid leukemia (CML). This study was performed for the first time to characterize the pharmacokinetics of radotinib, identify the factors contributing to pharmacokinetic variabilities and explore alternative dosing regimens. **Methods**: A total of 640 plasma concentration–time datapoints obtained from 47 participants were evaluated using nonlinear mixed-effects modeling to estimate pharmacokinetic parameters and evaluate covariate effects. The study population comprised 23 healthy volunteers (HVs) who received a single, oral dose of 400 mg radotinib and 24 CML patients who repeatedly received 300 mg twice daily. Based on the final population pharmacokinetic model, alternative dosing regimens to the current every 12 h regimen were explored using Monte Carlo simulations. **Results**: A two-compartment model with first-order absorption through transit compartments and first-order elimination incorporating a circadian rhythm effect best described radotinib pharmacokinetics. Disease status significantly affected apparent clearance; it was slower by 39.2% in CML patients compared with HVs (23.0 L/h versus 37.9 L/h), resulting in a longer terminal half-life (28.8 h versus 17.5 h). Age was negatively associated with volume of distribution in the central compartment, with an estimated slope of −0.0129 L/year. A 400 mg once-daily regimen was predicted to provide comparable systemic exposures to those of other TKIs with similar physiochemical and pharmacological properties to radotinib, and a 36% lower exposure than that of the current 300 mg twice-daily regimen. **Conclusions**: The model developed in this study adequately describes the population pharmacokinetics of radotinib and provides a basis for optimal, individualized radotinib therapy for patients with CML.

## 1. Introduction

Chronic myeloid leukemia (CML) is a myeloproliferative neoplasm characterized by the Philadelphia (Ph) chromosome arising from a reciprocal translocation of genes between chromosomes 9 and 22, which generates the breakpoint cluster region–Abelson tyrosine kinase 1 (BCR-ABL1) fusion gene that encodes a constitutively active tyrosine kinase driving uncontrolled myeloid proliferation [1,2,3,4]. CML presents in three phases—chronic, accelerated and blast—with the chronic phase accounting for approximately 90% of diagnoses and offering the most favorable prognosis when treated promptly [3,4]. Without effective therapy, CML progresses to accelerated and blast phases, underscoring the importance of early and effective intervention [3,4,5].

Radotinib (Supect^®^; IL-YANG Pharmaceuticals, Seoul, Republic of Korea) is an oral second-generation tyrosine kinase inhibitor (TKI) that has been used for treatment of CML in the chronic phase (CML-CP) in newly diagnosed patients and those who have resistance or intolerance to prior TKI therapy. Radotinib selectively targets and inhibits the BCR-ABL1 fusion protein [6,7,8,9], thereby effectively suppressing the proliferation of leukemic cells in Ph chromosome-positive (Ph+) CML-CP.

Clinical trials have demonstrated the great therapeutic effects of radotinib in CML-CP [10,11]. A phase 2 trial showed that radotinib was effective in patients with CML-CP who did not respond to previous TKIs [10]. Among 77 patients treated with 400 mg twice daily, 65% (*n* = 50) achieved a major cytogenetic response (MCyR), including 47% (*n* = 36) who attained a complete cytogenetic response (CCyR) at 12 months. The efficacy of radotinib was further supported by the phase 3 RERISE trial, in which radotinib demonstrated superiority over imatinib in achieving CCyR and major molecular response (MMR) in patients newly diagnosed with Ph+ CML-CP [11]. By 12 months, MMR was achieved in 54% of patients receiving 300 mg radotinib twice daily (37 of 69 patients), compared with 30% in the once-daily 400 mg imatinib group (20 of 66 patients; *p* = 0.0044).

Despite the favorable outcomes in radotinib therapy, the currently approved regimen of 300 mg twice daily under fasting conditions is often associated with tolerability concerns such as hepatotoxicity and adherence challenges arising from dietary restrictions [12,13]. The limitations highlight the need for dose individualization and optimization strategies of radotinib that maintain therapeutic efficacy while mitigating toxicity and enhancing compliance. Population pharmacokinetic (PK) modeling provides a rigorous, data-driven framework for optimizing a dosing regimen through the integration of individual characteristics. Population PK models characterize the concentration–time profile of a drug by quantifying its absorption, distribution, metabolism and excretion, and account for intra- and interindividual variabilities [14,15,16]. Population PK models not only characterize drug exposure from existing PK data but also explore better dosing strategies based on model-estimated parameters and relevant covariates [14,17,18,19]. Accordingly, the population PK modeling approach has been used to individualize drug dosing; facilitate dose optimization in special populations such as the elderly or those with organ impairment; and make regulatory decisions, including initial and revised labeling [17,18,20,21].

Hence, to fulfill the growing needs for dose individualization and optimization for radotinib therapy in patients with CML-CP, clinical PK studies were performed for the first time in healthy volunteers (HVs) and CML-CP patients. The nonlinear mixed-effects modeling approach was used to evaluate the PK characteristics, identify the factors that contribute to the PK variabilities and explore alternative dosing regimens for radotinib.

## 2. Results

### 2.1. Participant Characteristics

A total of 640 Cp datapoints of radotinib (306 Cp datapoints from 23 healthy volunteers and 334 Cp datapoints from 24 CML-CP patients) were evaluable for population PK modeling, obtained from 47 Asian participants, comprising 36 males (77%) and 11 females (23%) with a median (range) age of 31 (20–72) years (Table 1). The median (range) values of height, body weight and body mass index were 171 (155–186) cm, 65.7 (47.0–96.0) kg and 22.8 (18.4–30.3) kg/m^2^, respectively.

### 2.2. Bioanalytical Assay

Calibration curves for the two applied bioanalytical methods demonstrated linearity over the radotinib concentration range of 5 to 2000 ng/mL, with r^2^ values ≥ 0.999. The within-run and between-run accuracy and precision for all LLOQ, LQC, MQC and HQC samples met the acceptance criteria specified in the U.S. Food and Drug Administration guidance, namely accuracy within ±15% (±20% for LLOQ) and precision ≤ 15% (≤20% for LLOQ) [22].

In the validation of the bioanalytical method for plasma samples of healthy volunteers, within-run accuracy (% bias) for LLOQ, LQC, MQC and HQC samples ranged from −1.7% to 6.2%, −0.9% to 1.6%, −1.9% to 5.9% and −1.3% to 4.0%, respectively. Between-run accuracy was 2.3%, 0.7%, 1.1% and 0.5%, respectively. Within-run precision (% CV) for LLOQ, LQC, MQC and HQC samples ranged from 2.3% to 3.1%, 0.8% to 4.9%, 2.1% to 3.6% and 1.5% to 3.9%, respectively. Between-run precision was 4.1%, 3.0%, 4.3% and 3.6%, respectively. In the validation of the bioanalytical method for plasma samples of patients with CML-CP, within-run accuracy (% bias) for LLOQ, LQC, MQC and HQC samples ranged from −0.2% to 7.6%, −0.8% to 4.0%, 0.2% to 3.6% and 1.3% to 3.3%, respectively. Between-run accuracy was 4.6%, 2.4%, 2.4% and 2.0%, respectively. Within-run precision (% CV) for LLOQ, LQC, MQC and HQC samples ranged from 1.8% to 3.3%, 1.7% to 3.2%, 1.3% to 2.5% and 1.4% to 3.0%, respectively. Between-run precision was 4.1%, 3.3%, 2.5% and 2.3%, respectively.

### 2.3. Population Pharmacokinetic Model

The Cp–time curves of radotinib were best described by a two-compartment model incorporating transit compartments with first-order absorption as well as a first-order elimination process with the effects of circadian rhythm. The estimated parameters in the population PK model comprised CL/F, Vc/F, apparent intercompartmental clearance (Q/F), Vp/F, rate constant of first-order absorption (ka), mean transit time to absorption compartment (MTT) and the number of transit compartments (N). The variance of IIV was estimated for CL/F, while the variances for Vc/F, Q, Vp/F, ka, MTT and N were fixed at 0, 0, 0, 0, 0.1 and 0.2, respectively. No significant correlations were identified between IIVs for PK parameters. IOV was estimated for volumes of distribution (i.e., Vc/F and Vp/F). Residual variability was most appropriately described using a proportional model.

In the process of stepwise forward addition, disease status (i.e., CML-CP) and age were sequentially identified as the significant covariates associated with lower CL/F and smaller Vc/F, resulting in improved model fit with the ΔOFV of −8.7 and −12.8, respectively; disease status and age were incorporated into CL/F and Vc/F, respectively, as shown in the linear formula. All covariates included through the forward addition were retained in the final model following the process of backward elimination; each met the predefined retention criterion (ΔOFV > 6.63 upon elimination of each covariate). Hence, the final population PK model of radotinib (OFV = 6422) was as follows:

CL/F_i_ = 23.0 × [1 + 0.646 × (1 − CML_i_)] × [1 + 0.683 × cos{2π × (TIME − 7)/24}] × e^ηi^ (1)

Vc/F_i_ = 383 × [1 − 0.0129 × (AGE_i_ − 31)] × e^ηIOV^ (2)

Q/F_i_ = 132 (3)

Vp/F_i_ = 519 × e^ηIOV^ (4)

ka_i_ = 1.59 (5)

MTT_i_ = 1.88 × e^ηi^ (6)

N_i_ = 6.58 × e^ηi^ (7)

where CL/F_i_, Vc/F_i_, Q/F_i_, Vp/F_i_, ka_i_, MTT_i_ and N_i_ are the estimated CL/F, Vc/F, Q/F, Vp/F, ka, MTT and N of an individual participant; CML_i_ is the disease status of each individual participant (0 = HVs, 1 = patients with CML-CP); and AGE_i_ is the age of each individual participant at baseline (Table 2).

**Figure 1:**
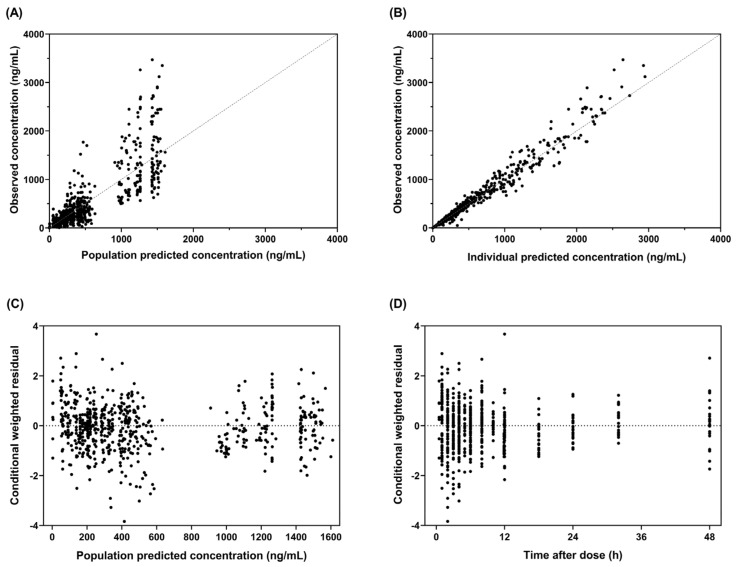
Goodness-of-fit plots for the final population pharmacokinetic model of radotinib: observed versus (**A**) population and (**B**) individual predicted concentrations; conditional weighted residuals versus (**C**) population-predicted concentrations and (**D**) time after dose.

**Figure 2:**
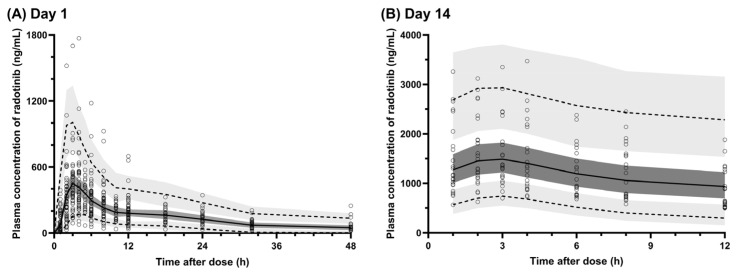
Visual predictive checks (VPCs) for the final population pharmacokinetic model of radotinib, overlaid with observed concentrations and stratified by sampling days: (**A**) VPC on Day 1 and (**B**) VPC on Day 14 [circles (O), observed concentrations; dashed lines (---), 5th and 95th percentile of predicted concentrations; solid lines, median of predicted concentrations; dark gray shaded areas, 95% confidence intervals of the median; light gray shaded areas, 95% confidence intervals of the predicted percentiles].

The final population PK model estimated all PK parameters of radotinib with adequate precision, as indicated by %RSE below 40% (Table 2). GOF plots illustrate a good agreement between predicted Cp from the final model and observed Cp (Figure 1A,B) with conditional weighted residuals randomly and symmetrically scattered around zero over the population-predicted Cp and time after dose (Figure 1C,D). VPCs for the final model, stratified by occasion (i.e., Days 1 and 14), show that more than 95% of the observed Cp fell within the 90% prediction intervals of the simulated data (Figure 2), demonstrating adequate predictive performance. The robustness of the model was further supported by comparable estimates of the PK parameters from a bootstrap with 1000 replicates to those from the final model (Table 2). Table 2 shows parameter estimates of the final population PK model for radotinib. The value of CL/F in patients with CML-CP was markedly lower than that in HVs. The estimated CL/F in HVs was lower by 39.2% than that in a typical patient with CML-CP (37.9 L/h versus 23.0 L/h). This difference was modeled with a linear disease effect coefficient of 0.646. For Vc/F, the estimated value in a participant at the median age of 31 years was 383 L with a negative linear effect of age on Vc/F (estimated slope: −0.0129 per year). Within the study population, over the ages of 20 to 72 years, Vc/F values were substantially varied, ranging from 180 to 437 L.

### 2.4. Exploration of Alternative Dosing Regimens Using Monte Carlo Simulations

**Figure 3:**
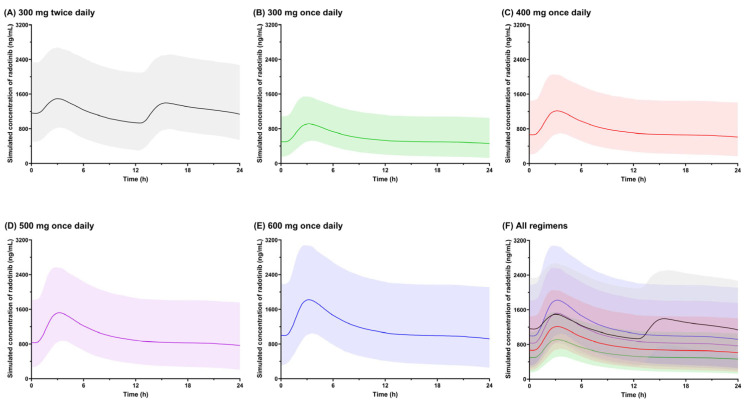
Simulated concentration–time profiles of radotinib at steady state in virtual patients with chronic myeloid leukemia (*n* = 2400 per group) following administrations of the following: (**A**) 300 mg twice daily, (**B**) 300 mg once daily, (**C**) 400 mg once daily, (**D**) 500 mg once daily, (**E**) 600 mg once daily and (**F**) all regimens combined. Profiles are presented as median values with 90% prediction intervals. [Colored lines represent the median simulated concentrations for each dosing regimen (gray, green, red, purple and blue for panels (**A**)–(**E**), respectively), and the corresponding shaded areas indicate the 90% prediction intervals].

The median and 90% prediction intervals of the simulated Cp–time profiles for radotinib at steady state under once- or twice-daily dosing regimens are presented in Figure 3. Upon administration, predicted Cp increased with a delay of approximately 0.5 h, reached C_max_ in approximately 3 h and declined until the subsequent dose was administered. In 2400 virtual patients receiving 300 mg radotinib twice daily, the median (interquartile range, IQR) simulated values of AUC_0–24h_, C_max_ and C_trough_ at steady state were 29,669 (22,710–38,990) ng∙h/mL, 1551 (1234–1959) ng/mL and 960 (645–1368) ng/mL, respectively (Table 3). At the radotinib dosing regimen of 400 mg once daily, the corresponding values were 19,034 (14,169–25,645) ng∙h/mL, 1274 (1011–1564) ng/mL and 591 (396–877) ng/mL, respectively. Compared with the currently approved dosing regimen of 300 mg twice daily, 400 mg once daily resulted in a 36% reduction in AUC_0–24h_, 18% in C_max_ and 38% in C_trough_.

## 3. Discussion

This study was conducted to determine the pharmacokinetic characteristics of radotinib and identify the factors that contribute to its pharmacokinetic variabilities using the concentration–time data obtained from healthy volunteers and patients with CML-CP through nonlinear mixed-effects modeling. The population PK characteristics of radotinib were adequately depicted by a two-compartment model incorporating transit compartments with first-order absorption and first-order elimination influenced by circadian rhythm. The model was adequately performed as evidenced by multiple diagnostic assessments including GOF plots (Figure 1), VPC plots (Figure 2) and nonparametric bootstrap resampling (Table 2). The final model developed in this study provides insights into the PK of radotinib and will likely serve as a basis for data-informed optimization of precision radotinib therapy in practice.

The population estimates of CL/F were 37.9 L/h and 23.0 L/h in HVs and patients with CML-CP, respectively (Table 2). Our estimated CL/F in HVs was slightly slower than the preliminarily assessed value of 51.0 L/h that was calculated from dose/AUC_0–∞_ using noncompartmental analysis (NCA) [23]. This discrepancy in CL/F between the model-derived and preliminary values appeared to result from the methodological differences in computing CL/F between nonlinear mixed-effects modeling and NCA. Regarding distribution, the population estimates of Vc/F and Vp/F were 383 L and 519 L, respectively, leading to a total V/F (i.e., summation of Vc/F and Vp/F) of 902 L (Table 2). Our estimated V/F was slightly smaller than the preliminarily evaluated V/F of 1315 L in HVs based on NCA [23]. This difference seems to be attributed to the effects of age on Vc/F in our population PK model as well as a different computing method of nonlinear mixed-effects modeling in our present study. For terminal half-lives (t_1/2_), the population estimates were 18.2 h and 28.8 h in HVs and patients with CML-CP, respectively. The model-estimated t_1/2_ were consistent with the preliminary values of 17.5 h and 25.4 h in HVs and patients with CML-CP, respectively [23,24].

Following repeated administrations of 300 mg radotinib twice daily to patients with CML-CP, the median (IQR) values of simulated C_max_, time to C_max_ (t_max_) and AUC_0–24h_ at steady state were 1551 (1234–1959) ng/mL, 3 (2–3) h and 29,669 (22,710–38,990) ng∙h/mL, respectively (Table 3). The simulated values of C_max_ and t_max_ in patients with CML-CP were consistent with the preliminarily assessed values of 1595 (1143–2393) ng/mL and 2.5 (2.0–3.8) h, respectively [24]. The predicted value of AUC_0–24h_ was also comparable to the previously estimated AUC_0–24h_ of 23,968 (19,314–42,326) ng·h/mL that was obtained by doubling the preliminary value of AUC_0–12h_. Collectively, these findings indicate good agreement of the simulated and the observed exposure in patients with CML-CP.

Among tested covariates, CML-CP was identified as a significant covariate affecting the CL/F of radotinib; patients with CML-CP exhibited a CL/F that was slower than HVs by 39.2%, as captured by a coefficient of 0.646 in the final population PK model (Table 2). This slower CL/F led to a prolonged t_1/2_ in patients with CML-CP compared to that in HVs (28.8 h versus 18.2 h). One plausible explanation is the chronic inflammatory state associated with CML [25,26,27], characterized by elevated pro-inflammatory cytokines such as interleukin-6 (IL-6) and tumor necrosis factor-alpha (TNF-α) that are known to suppress CYP3A expression and activity [28,29,30,31]. Given that radotinib, like other TKIs, is primarily metabolized by CYP3A4 [32,33,34,35], such suppression likely reduces metabolic clearance and increases the bioavailability of radotinib due to a decreased first-pass effect during absorption, even in the absence of clinically apparent hepatic dysfunction. Another plausible explanation is altered plasma protein binding. In CML, elevated α_1_-acid glycoprotein levels potentially decrease the unbound fraction (f_u_) of radotinib [36,37]. Given that radotinib shares structural similarities with nilotinib and imatinib, which are drugs with a low hepatic extraction ratio [38], a reduction in f_u_ would be expected to decrease hepatic clearance accordingly [39,40]. The other plausible explanation for the slower CL/F in CML patients is the drug–drug interactions between radotinib and concomitant medications that are frequently used to manage comorbidities in patients with CML-CP [41,42]. In particular, concomitant medications that inhibit CYP3A4 would reduce the CL/F of radotinib. This finding suggests that the PK characteristics of radotinib obtained from HVs may not be directly applicable to patients with CML-CP, highlighting the importance of utilizing data from patients with CML-CP (i.e., the target disease) in model-informed optimization of radotinib dosing regimens.

Age was identified as a significant covariate that influenced the Vc/F of radotinib with a negative linear relationship (−0.0129 L per year) (Table 2). This age-related decline in Vc/F appears to be attributed to physiological changes associated with aging rather than age itself. A possible explanation lies in age-related changes in body composition such as increased fat mass as well as reduced lean body mass and total body water, all of which are known to affect the distribution of systemically administered drugs [43,44]. Notably, such changes may occur even when total body weight remains unchanged [45,46]. Although statistically significant, the clinical magnitude of the age effect appears limited within the age range of this study (20–72 years). Given that Vc/F primarily influences the distribution phase rather than steady-state exposure, which is determined by clearance, the observed age-related reduction in Vc/F is unlikely to warrant dose adjustment in typical clinical practice. However, this effect may become clinically relevant in very elderly individuals (e.g., >80 years), although such patients were not included in this study.

In the final population PK model, the effect of circadian rhythm (coefficient of the effect = 0.683) was integrated into the elimination process (i.e., CL/F) to account for diurnal fluctuations in the metabolism of radotinib (Table 2). Such an effect is plausibly linked to evidence demonstrating that the expression and activity of hepatic enzymes (e.g., cytochrome P450 family) and/or transporters fluctuate over the circadian cycle, thereby modulating the CL/F of a drug throughout the day [47,48,49]. Similar circadian variations in pharmacokinetics have been reported for various chronopharmacologically sensitive agents such as tacrolimus, 5-fluorouracil and doxorubicin [50,51,52]. In particular, CYP3A activity, which is primarily responsible for the metabolism of radotinib, has been reported to peak at approximately 15:00 in humans [47], in close agreement with 16:00, the time at which the model estimated the CL/F of radotinib to reach its peak activity. Therefore, our circadian-modulated model is suggested to be biologically plausible, underscoring the significance of circadian effects in PK characteristics.

Given that the t_1/2_ of radotinib in patients with CML-CP is estimated to be 28.8 h, once-daily dosing might be suggested as a pharmacokinetically and clinically relevant alternative regimen to the currently approved 300 mg twice-daily regimen. This t_1/2_ is notably longer than that of imatinib (18 h) [35] and nilotinib (16 h) [53]. Despite the t_1/2_ of imatinib being shorter than that of radotinib, once-daily administration of imatinib has been shown to achieve sufficient therapeutic efficacy, supporting the feasibility of a once-daily regimen for radotinib as well.

Higher systemic exposure to radotinib has previously been linked to a higher probability of dose-limiting toxicities, which lead to treatment interruptions or dose reductions often associated with compromised long-term efficacy [12,13]. Moreover, the currently approved regimen of 300 mg twice daily under fasting conditions is often accompanied by tolerability concerns, such as hepatotoxicity, and poses challenges to adherence owing to dietary restrictions. A once-daily regimen may therefore offer a simplified treatment alternative that improves tolerability and adherence. As a representative potential once-daily regimen, 400 mg was predicted to reduce AUC_0–24h_ by 36%, C_max_ by 18%, and C_trough_ by 38% compared with the current regimen based on Monte Carlo simulations (Table 3). The predicted median C_trough_ of 591 ng/mL was comparable to the target C_trough_ of 500 ng/mL when given 400 mg of nilotinib once daily, which is a second-generation TKI with structural similarity to radotinib, in terms of favorable molecular responses [54]. Collectively, these findings provide a pharmacokinetic basis for clinical evaluation of once-daily regimens of radotinib. Further prospective studies evaluating 400 mg once-daily dosing, along with characterization of the exposure–response relationship, will be essential to validate these predictions and enable model-informed dose optimization.

This study has some limitations to consider. First, the model was developed using data from a relatively small cohort of 47 participants, all of whom were of Asian descent. Consequently, the effect of ethnicity on the PK of radotinib remains unknown. Further studies are being planned to evaluate this effect in larger and more ethnically diverse populations. Second, variabilities remained between observed and population-predicted concentrations (Figure 1A), particularly on Day 14 (i.e., steady state), for which data from only 24 patients with CML-CP were available. The large interindividual and unexplained variability may be attributed to the limited sample size, heterogeneity in clinical status among participants and unexplored covariates such as genetic polymorphisms and transporter-mediated processes, which warrant further investigation. Third, the effects of concomitant medications, particularly those that modulate CYP3A4 activity, were not assessed as independent covariates but were implicitly captured by a disease status variable. Additional research is also warranted to evaluate the specific impact of drug–drug interactions on the PK of radotinib. Fourth, the apparent effect of disease status on CL/F may be confounded by demographic differences between study cohorts. Healthy volunteers consisted exclusively of Korean males, whereas patients with CML-CP included both sexes and only Chinese participants. As such, the observed effect may, in part, reflect interethnic variability or sex-related differences rather than an effect of disease status itself. Further studies are needed to clarify the impact of these potential confounding factors.

## 4. Materials and Methods

### 4.1. Study Participants and Design

Two clinical PK studies of radotinib were performed in healthy volunteers and patients with CML-CP. The two studies were approved by the institutional review board (IRB) of Severance Hospital and Peking University People’s Hospital (IRB numbers 4-2024-0489 and 2018PHA053, respectively) and were conducted in accordance with the Declaration of Helsinki and the International Council for Harmonization Good Clinical Practice (ICH-GCP) guidelines [55]. All participants provided written informed consent prior to any study-related procedures. The studies were registered at ClinicalTrials.gov (identifiers: NCT06461078 and NCT03722420). The present population PK study was performed using pooled data from two clinical studies.

The first study (NCT06461078) included healthy male adult volunteers aged 19 to 55 years, weighing 55 kg or heavier, with a body mass index (BMI) between 18.5 kg/m^2^ and 27.0 kg/m^2^. Individuals with a history of clinically significant psychiatric or comorbid conditions such as severe hepatic impairment, severe renal impartment, long QT syndrome; a history of gastrointestinal disorders or surgeries that may have affected the absorption of radotinib; or suspected symptoms of acute illness at the time of screening were excluded. Also excluded were individuals with concomitant medications or foods that may have interfered with the study outcomes as well as those with excessive consumption of caffeine, alcohol or tobacco. Individuals were also ineligible if they had participated in other clinical trials within 180 days, donated whole blood within 60 days or blood components within 30 days or received a blood transfusion within 30 days prior to the administration of radotinib. Study participants received a single oral dose of 400 mg radotinib under fasting conditions. Serial blood samples were collected from forearm veins of participants pre-dose and at 0.5, 1, 2, 3, 4, 5, 6, 8, 10, 12, 18, 24, 32 and 48 h post-dose.

The second study (NCT03722420) included patients aged 18 years or older with a confirmed diagnosis of Ph+ CML-CP within last 6 months, typical *BCR-ABL1* transcript type and normal organ function. Patients with a T315I mutation in the *BCR-ABL1* gene; a history of radiotherapy, interferon or other targeted anti-leukemic therapies; or clinically significant comorbidities were excluded. Patients were also ineligible if they had participated in other investigational studies within the past 3 months or were taking therapeutic coumarin derivatives; a moderate or strong CYP3A4 inhibitor or inducer; or medications that may have prolonged the QT interval. Study participants were orally administered 300 mg radotinib twice daily under fasting conditions. Serial blood samples were obtained from forearm veins pre-dose and at 1, 2, 3, 4, 6, 8 and 12 h post-dose on Days 1 and 14.

### 4.2. Bioanalytical Assay and Validation

All samples were centrifuged to separate plasma and stored at −70 °C until the measurement of plasma concentrations (Cp) of radotinib. The Cp of radotinib in HVs was measured using high-performance liquid chromatography coupled with tandem mass spectrometric detection (HPLC-MS/MS; Prominence HPLC/UFLC/UFLCXR, Shimadzu, Kyoto, Japan; QTRAP 6500+, SCIEX, Marlborough, MA, USA). An internal standard was radotinib-d_6_ dissolved in acetonitrile to produce working solutions. The linearity of the bioanalytical method was assessed using a calibration curve computed by a weighted (1/x^2^) linear regression from seven standard concentrations (5, 10, 30, 100, 300, 1000 and 2000 ng/mL). The method was validated using concentrations of 5, 15, 160 and 1600 ng/mL for the lower limit of quantitation (LLOQ) and low- (LQC), medium- (MQC) and high-quality controls (HQC), respectively.

In patients with CML-CP, the Cp of radotinib was quantified using another HPLC–MS/MS system (LC-20ADXR pump, CTO-20AC column oven, CBM-20A Lite controller and DGU-20A5R degasser, Shimadzu, Kyoto, Japan; API 4000, SCIEX, MA, USA). Glipizide in acetonitrile was used as an internal standard. Linearity was evaluated using a calibration curve constructed by a weighted (1/x^2^) linear regression from eight standard concentrations (5, 10, 30, 100, 300, 1000, 1600 and 2000 ng/mL). The method was validated using LLOQ, LQC, MQC and HQC concentrations of 5, 12.5, 500 and 1500 ng/mL, respectively. For both bioanalytical methods, accuracy and precision were determined by calculating relative bias (% bias) and the coefficient of variation (% CV), respectively.

### 4.3. Population Pharmacokinetic Characterization

The population PK analysis of radotinib was performed using a nonlinear mixed-effect modeling approach implemented in NONMEM^®^ software (version 7.5.1; ICON Development Solution, Ellicott City, MD, USA). All model parameter values were estimated using the first-order conditional estimation method with interactions [56].

One- and two-compartment models incorporating first-order absorption and elimination were tested as initial structural PK models. The absorption phase was further characterized using various models such as a first-order absorption model, a mixed first-order absorption model with parallel zero-order absorption, a zero-order absorption model, models with lag time for first- and zero-order absorption and a transit compartment model with first-order absorption. Elimination from the central compartment was modeled as a first-order process, as nonlinear elimination models did not improve the model fit. Interindividual variability (IIV, η) of PK parameters was modeled assuming a log-normal distribution (mean = 0, variance = ω_1_^2^), with potential correlations assessed through the OMEGA BLOCK function [57]. Residual variability (ε) was assumed to follow a normal distribution (mean = 0, variance = σ^2^), and was evaluated using additive, proportional or combined error models. Interoccasion variability (IOV) was incorporated in the model assuming a log-normal distribution (mean = 0, variance = ω_2_^2^) [58,59], where each occasion was defined as a distinct pharmacokinetic state (i.e., Day 1 as non-steady state, Day 14 as steady state). The effects of circadian rhythm on PK parameters were evaluated as IOV using a cosine function with amplitude and phase shift to capture rhythmic fluctuations over a 24 h cycle [60]. The best structural model was selected based on the objective function value (OFV), Akaike information criterion (AIC), goodness-of-fit (GOF) plots, relative standard errors (RSEs) and visual inspection of individual concentration–time profiles overlaid with observed data. All diagnostic tests and graphical analyses were performed using Xpose4 in R software (version 4.4.2; R project, http://www.r-project.org; accessed 25 August 2025) [61].

After establishing the base PK model, the final PK model was developed by evaluating the impact of covariates on key PK parameters, including apparent clearance (CL/F), apparent volume of distribution (V/F) of the central compartment (Vc/F) and V/F of the peripheral compartment (Vp/F). Evaluated covariates were disease status (i.e., healthy volunteers = 0 and CML patients = 1), demographic factors (e.g., age, sex and body weight), hepatic function (e.g., alanine aminotransferase and aspartate aminotransferase), renal function (creatinine clearance [CL_Cr_]) and other clinical laboratory measures such as plasma protein concentrations. Body surface area and CL_Cr_ were calculated using the Mosteller formula and Cockcroft–Gault equation, respectively [62,63]. Prior to covariate modeling, the relationship between each covariate and PK parameter was assessed for physiological plausibility. Continuous covariates (e.g., body weight) were tested using linear, exponential or power functions, whereas categorical covariates (e.g., disease status) were modeled using additive or proportional functions. The statistical significance of covariates was evaluated using automated stepwise covariate modeling in Perl-speaks-NONMEM (PsN) version 5.5.0, based on the changes (Δ) in OFVs, with selection criteria of a decrease in OFV greater than 3.84 (*p* < 0.05) for forward addition and an increase in OFV greater than 6.63 (*p* < 0.01) for backward elimination [64].

The robustness of the final model was evaluated through a combination of graphical and statistical approaches, including GOF plots, visual predictive checks (VPCs) and the nonparametric bootstrap resampling method. Model fit to the observed data was assessed by visual inspection of GOF plots. The predictive performance of the final model was examined using VPC plots generated from 1000 simulations stratified by occasion. For visual comparison, the observed Cp–time data were plotted alongside the medians and 90% prediction intervals of the simulated profiles. From the original dataset, 1000 replicated datasets were generated using a nonparametric bootstrap resampling method implemented in Wings for NONMEM (version 751; http://wfn.sourceforge.net; accessed 25 August 2025). Each replicated dataset retained the same number of HVs and CML patients as the original one, but with a different number of observations. The median values and corresponding 95% confidence intervals (CIs) of the bootstrap-estimated PK parameters were then compared with the final model estimates.

All statistical analyses not otherwise specified, as well as the generation of graphical figures, were performed using GraphPad Prism (version 10.5.0; GraphPad Software Inc., Boston, MA, USA).

### 4.4. Exploration of Dosing Regimen Using Monte Carlo Simulations

Alternative dosing regimens of radotinib were explored at the following regimens: 300 mg twice daily and 300, 400, 500 and 600 mg once daily. Plasma concentration–time profiles of radotinib at steady state for each regimen were generated through Monte Carlo simulations using NONMEM. For each regimen, virtual populations (*n* = 4700; 100 replicates) were produced by keeping the same distribution of baseline characteristics as that for the population used for model development. Simulated data corresponding to patients with CML-CP were extracted for further analysis. Systemic exposures of radotinib, including maximum concentrations (C_max_), trough concentrations (C_trough_) and area under the concentration–time curve (AUC) over 24 h (AUC_0–24h_), were calculated using simulated plasma concentration–time profiles.

## 5. Conclusions

This is the first study to characterize the pharmacokinetics of radotinib and identify the factors that contribute to the pharmacokinetic variabilities in healthy volunteers as well as patients with chronic myeloid leukemia. Based on nonlinear mixed-effects modeling, the two-compartment model with first-order absorption via transit compartments and first-order elimination incorporating the effect of circadian rhythm adequately describes the population pharmacokinetics of radotinib. Disease status and age significantly affect the pharmacokinetics of radotinib, with slower CL/F in CML-CP patients and smaller Vc/F in older individuals. The markedly slower CL/F in patients leads to a prolonged terminal half-life of 28.8 h, indicating the current 12 h dosing interval might need to be extended. The findings in this population PK study potentially provide a basis for suggesting optimal, individualized dosing regimens of radotinib in clinical settings.

## Figures and Tables

**Table 1:** Demographics and baseline characteristics of study participants.

	Healthy Volunteers (*n* = 23)	Patients with CML (*n* = 24)	Total (*n* = 47)
Sex, n (%)			
Male	23 (100)	13 (54)	36 (77)
Female	0 (0)	11 (46)	11 (23)
Age, years			
Median (range)	29 (20–51)	32 (21–72)	31 (20–72)
Height, cm			
Median (range)	173 (166–186)	168 (155–178)	171 (155–186)
Body weight, kg			
Median (range)	71.0 (55.1–85.2)	65.0 (47.0–96.0)	65.7 (47.0–96.0)
BMI, kg/m^2^			
Median (range)	22.8 (18.6–26.2)	22.5 (18.4–30.3)	22.8 (18.4–30.3)
Ethnicity, n (%)			
Korean	23 (100)		23 (49)
Chinese		24 (100)	24 (51)
ALT, IU/L			
Median (range)	16 (7–80)	21 (15–75)	20 (7–80)
AST, IU/L			
Median (range)	19 (11–76)	25 (15–41)	20 (11–76)
CL_Cr_, mL/min			
Median (range)	117 (72–139)	109 (74–200)	114 (72–200)

ALT, alanine aminotransferase; AST, aspartate aminotransferase; BMI, body mass index; CML, chronic myeloid leukemia; CL_Cr_, creatinine clearance.

**Table 2:** Parameter estimates of radotinib from the final population pharmacokinetic model and the bootstrap of 1000 replicates.

	Population Estimates	RSE %	Bootstrap Estimate Median (95% CI)
Model parameters			
CL/F (L/h) ^a^	23.0	7.3	23.2 (20.0–26.5)
CL/F_Circadian effect_	0.683	14.8	0.686 (0.502–0.871)
CL/F_Disease status_	0.646	30.0	0.625 (0.249–1.02)
Vc/F (L) ^b^	383	13.8	395 (281–526)
Vc/F_Age_	−0.0129	37.6	−0.0127 (−0.0258–−0.003)
Q/F (L/h)	132	11.6	126 (88.9–185)
Vp/F (L)	519	15.6	529 (398–733)
ka (h^−1^)	1.59	21.6	1.56 (1.07–3.14)
MTT (h)	1.88	10.4	1.86 (1.43–2.27)
N	6.58	17.0	6.34 (4.46–10.6)
Interindividual variability			
ω_CL/F_	0.389	20.1	0.379 (0.301–0.459)
ω_Vc/F_	0	NA	0
ω_Q/F_	0	NA	0
ω_Vp/F_	0	NA	0
ω_ka_	0	NA	0
ω_MTT_	0.316	NA	0.316
ω_N_	0.447	NA	0.447
Interoccasion variability			
ω_IOV_	0.698	25.1	0.669 (0.494–0.829)
Residual variability			
σ_proportional_ (%)	20.0	NA	20.0

CL/F, apparent clearance; Vc/F, apparent volume of distribution of the central compartment; Vp/F, apparent volume of distribution of the peripheral compartment; Q/F, apparent intercompartment clearance; CI, confidence interval; ka, first-order rate constant; ωCL/F, interindividual variability of apparent clearance; ωMTT, interindividual variability of the mean transit time; ωN, interindividual variability of the number of transit compartments; ωIOV, interoccasion variability; MTT, mean transit time; NA, not applicable; N, number of transit compartments; σproportional, proportional residual error; RSE, relative standard error. ^a^ Final model of CL/F (L/h)  =  23.0 × [1 + 0.646 × (1 − disease status)] × [1 + 0.683 × (circadian effect)]; disease status: 0 = healthy volunteers, 1 = patients with chronic myeloid leukemia. ^b^ Final model of Vc/F (L) = 383 × [1 − 0.0129 × (age − 31)].

**Table 3:** Simulated steady-state systemic exposures of radotinib at various dosing regimens. Values are presented as median (interquartile range).

Parameter (Units)	300 mg BID	300 mg QD	400 mg QD	500 mg QD	600 mg QD
t_max_ (h)	3 (2–3)	3 (3–4)	3 (3–4)	3 (3–4)	3 (3–4)
C_max_ (ng/mL)	1551 (1234–1959)	955 (758–1173)	1274 (1011–1564)	1592 (1264–1955)	1910 (1516–2345)
C_trough_ (ng/mL)	960 (645–1368)	443 (297–658)	591 (396–877)	739 (495–1097)	887 (594–1316)
AUC_0–24h_ (ng∙h/mL)	29,669 (22,710–38,990)	14,276 (10,627–19,234)	19,034 (14,169–25,645)	23,793 (17,711–32,056)	28,551 (21,253–38,467)

AUC_0–24h_, area under the concentration–time curve from 0 to 24 h; C_max_, maximum concentration; QD, once-daily dosing; C_trough_, trough concentration; t_max_, time to maximum concentration; BID, twice-daily dosing.

## Data Availability

Data are available from the corresponding author only upon reasonable request due to privacy/ethical restrictions.
